# Supplementary material for: SARS-CoV-2 human challenge reveals biomarkers that discriminate early and late phases of respiratory viral infections
Source: Nat Commun. 2024 Nov 30;15:10434. doi: 10.1038/s41467-024-54764-3 (PMC11608262; doi:10.1038/s41467-024-54764-3)
Supplement: Supplementary file 1 — Supplementary Information [file 41467_2024_54764_MOESM1_ESM.pdf]

Supplementary Figures

Contents

Supplementary Table 1 ... 2

Characteristics of whole-blood RNA signatures for viral infection included in analysis ... 2

Supplementary Figure 1 ... 7

Identification of blood transcriptional biomarkers of viral infection by systematic review and comparison of their gene composition. ... 7

Supplementary Figure 2 ... 8

Principal component analysis of RNA sequencing data ... 8

Supplementary Figure 3 ... 9

Temporal profiles of blood transcriptional signature scores in participants with and without replicative SARS-CoV-2 infection. ... 9

Supplementary Figure 4 ... 10

Discrimination of participants with and without replicative SARS-CoV-2 infection by blood transcriptional biomarkers of viral infection. ... 10

Supplementary Figure 5. ... 11

Relationship of MX1 and IFI27 blood RNA levels with time from virus challenge in replicative SARS-CoV-2 infection. ... 11

Supplementary Figure 6 ... 12

Blood MX1 and IFI27 blood transcript correlation with PCR viral load measurements. ... 12

Supplementary Figure 7 ... 13

ATAC sequencing reads at *MX1* and *IFI27* locus in unstimulated peripheral blood mononuclear cells. ... 13

Supplementary Figure 8. ... 14

Blood transcript discrimination of post infection time points from pre-infection samples in in unselected community acquired respiratory virus infections from GSE68310. ... 14

Supplementary Table 1

Characteristics of whole-blood RNA signatures for viral infection included in analysis

Signature(s)	Model¶	Discovery population(s)	Discovery setting(s)	Discovery approach	Validation population(s)	Intended application
**AndresTerres11 (1)**	Geometric mean of all genes (influenza meta-signature)	Five cohorts of children and adults with influenza; adults challenged with influenza; and adults with bacterial pneumonia	UK, USA, and Australia	Differential expression followed by leave-one-cohort-out strategy and filtering for heterogeneity of effect size, using genome-wide data	Eight cohorts of children or adults with influenza or bacterial infection; adults challenged with influenza; and adults vaccinated against influenza	Influenza *vs* bacterial or other viral infection
**Cappuccio11(2)**	Difference in geometric means between upregulated and downregulated genes (COVID-19 Signature)	84 publicly available transcriptomic studies classified into four categories: COVID-19 contrasts; other viral infection (respiratory and non-respiratory) contrasts; bacterial infection (respiratory and non-respiratory) contrasts; non-infectious contrasts. A typical contrast included samples from diseased subjects and healthy controls	Worldwide	Multi-objective fitness function that evaluates any proposed signature along three dimensions: detection, consistency with ATAC-seq and pathway annotation, and cross-reactivity. The multi-objective fitness function was optimized in training studies to return a population of high-fitness candidate signatures which were further selected based on performance in a set of development studies. The signature showing the most consistent performance in both training and development studies was selected.	43 publicly available transcriptomic studies classified into four categories: COVID-19 contrasts; other viral infection (respiratory and non-respiratory) contrasts; bacterial infection (respiratory and non-respiratory) contrasts; non-infectious contrasts. A typical contrast included samples from diseased subjects and healthy controls	COVID-19 infection *vs* healthy, other bacterial/viral infections and non-infectious conditions
**Gómez-Carballa3 (3)**	Logistic regression§	Aggregated data from 11 publicly available cohorts containing children and adults with viral or bacterial infections	USA, UK, Spain, The Netherlands, Australia, Mexico	Parallel Regularized Regression Model Search (4) on 64 candidate genes previously reported in the 11 cohorts to distinguish viral from bacterial infection	Split test cohort of independent samples from the aggregated dataset of 11 publicly available cohorts containing children and adults with viral or bacterial infections	Viral *vs* bacterial infection
**Henrickson16 (5)**	Difference in geometric means between upregulated and downregulated genes (influenza paediatric signature score)	Four cohorts of children with influenza-like illness	USA	Meta-analysis and leave-one-out strategy to identify common genes using genome-wide data	Two cohorts of children or adults with influenza	Influenza infection *vs* healthy

**Herberg2 (6)**	Sum of downregulated genes subtracted from the sum of upregulated genes (Disease Risk Score)	Children with viral or bacterial infection	UK, USA, and Spain	Elastic net followed by forward selection–partial least squares, using significantly differentially expressed transcripts	Children with bacterial or viral infection, inflammatory disease, or indeterminate diagnosis	Viral *vs* bacterial infection in febrile children
**IFI27 (7)[#]**	NA	Two cohorts of adults with influenza & one cohort of adults with influenza, bacterial infection, non-infectious and healthy controls	Australia, Canada, Germany	Differential gene expression between influenza cases and healthy control. IFI27 selected based on fold change and adjusted p value	Four publicly available cohorts of adults/children with viral infection, bacterial infection, non-infectious and healthy controls. One prospective cohort of adults presenting with suspected respiratory tract infection	Influenza *vs* bacterial infection
**IFI44L (9)**	NA	Children with viral or bacterial infection (6)	UK, USA and Spain	Elastic net followed by forward selection–partial least squares, using significantly differentially expressed transcripts	Children with bacterial or viral infection	Viral *vs* bacterial infection in febrile children
**IFIT3; RSAD2* (10)**	NA	Three cohorts of adults challenged with rhinovirus, influenza or RSV (11)	UK and USA	Sparse latent factor regression analysis on genome-wide data (11) followed by regularised logistic regression on the resulting 30-gene signature	Close contacts of students with acute upper respiratory viral infections	Pre-symptomatic viral infection *vs* healthy
**Li3 (12)**	Logistic regression (FS-PLS signature)§	Adults with confirmed diagnosis of viral infection, bacterial infection or no infection	UK	Forward selection–partial least squares method (6) (13) applied to differentially expressed genes between definite bacterial and definite viral groups	One cohort of adults with definite viral, definite bacterial, probable viral, probable bacterial, indeterminate infection and non-infected/ other infection (e.g. fungal)<br>One cohort of adults with COVID-19 or confirmed bacteraemia	Viral (including COVID-19) *vs* bacterial
**Lopez7 (14)**	Sum of weighted gene expression values (bacterial *vs* viral classifier)	Children and adults with viral, bacterial, or non-infectious acute respiratory illness (15)	USA	Support vector machine analysis using genome-wide data	Children with acute viral or bacterial infections (16)	Viral *vs* bacterial respiratory infection
**Lydon15 (17)**	Logistic regression (Viral classifier)§	Adolescents and adults with viral, bacterial, or non-infectious acute respiratory illness	USA	LASSO regression analysis using 87 selected target genes from previously derived signatures (15,18)	Patients with viral or bacterial co-infection or suspected bacterial infection	Viral *vs* bacterial respiratory infection
**MX1 (19)**	NA	NA	NA	Pre-selected due to biological plausibility	Adults challenged with the live yellow fever virus vaccine	Viral infection *vs* healthy

**Pennisi2 (20)**	Sum of downregulated genes subtracted from the sum of upregulated genes	Children with viral or bacterial infection (6)	UK, USA and Spain	Elastic net followed by forward selection–partial least squares, using significantly differentially expressed transcripts (6), then selection of an adequately expressed transcript for use in RT-LAMP	Children with bacterial or viral infection	Viral *vs* bacterial infection in children
**Rao8 (21)**	Difference in geometric means between genes upregulated in bacterial infection & genes upregulated in viral infection (BoVI Score)	Aggregated data from 32 publicly available cohorts containing subjects with viral infection, bacterial infection and healthy controls	Worldwide	Greedy backward search and abridged best subset selection performed on 100 genes with the highest scores in SAM analysis with LOSO analysis	Retrospective validation in aggregated data from 32 publicly available cohorts containing subjects with viral infection, bacterial infection and healthy controls and five individual cohorts containing subjects with viral infection and bacterial infection Prospective validation in two cohorts of febrile adults & children with bacterial and viral infection	Viral *vs* bacterial infections
**Ravichandran10 (22)**	Difference in geometric means between genes upregulated in bacterial infection and those downregulated in bacterial infection/upregulated in viral infection (VB_{10})	Aggregated data from six publicly available cohorts containing subjects with viral infection, bacterial infection and healthy controls	UK, USA, Spain	Condition specific response networks computed from differentially expressed genes with network mining to identify top ranked perturbations from which top genes are selected based on a statistical threshold for differential gene expression across all discovery datasets	Aggregated data from 50 publicly available cohorts containing subjects with viral infection, bacterial infection and healthy controls<br><br>Bangalore – Viral Bacterial (BL-VB) cohort of adults with bacterial, viral and indeterminate infection and healthy controls	Viral *vs* bacterial infections
**RRM2(23)**	NA	Adults hospitalised with respiratory illness who tested positive or negative for COVID-19(24)	USA	Network analysis applied to differentially expressed genes and Maximal Clique Centrality (MCC) algorithm to identify top-ranked nodes	One cohort of adults with COVID-19 and healthy controls	COVID-19 *vs* healthy
**Sampson10 (25)**	SeptiCyte™ TRIAGE score (25) minus SeptiCyte™ VIRUS score (26) (Combined SeptiCyte score)	Eight cohorts of neonates, children, and adults with bacterial infections	UK, USA, Estonia and Australia	Regression analysis of transcript pairs using the 6000 most highly expressed genes from each dataset	Unselected consecutive patients presenting to the emergency department with febrile illness	Viral *vs* bacterial in febrile patients
**Sampson4 (26)**	Linear sum of upregulated and downregulated	Ten cohorts of children and adults with viral infections; two cohorts	USA, Brazil, Finland and Australia	Regression analysis of transcript pairs using the 6000 most highly expressed genes from each dataset	Seven human cohorts and six non-human mammal cohorts infected or challenged with	Viral *vs* non-viral conditions

	transcripts (Septicyte VIRUS)	of adults challenged with influenza; and two cohorts of macaques challenged with Lassa virus or lymphocytic choriomeningitis virus			viruses across all seven of the Baltimore virus classification groups	
**Steinbrink19[‡] (27)**	Logistic regression§	Patients with candidaemia, viral infection, bacterial infection, or non-infectious SIRS and healthy controls	USA	Regularized multinomial logistic regression (LASSO) with nested leave one sample out cross-validation performed on differentially expressed genes identified by generalized linear hypothesis testing	Three cohorts: adults with candidaemia, viral infection, bacterial infection and healthy controls; children and adults with viral, bacterial or non-infectious acute respiratory illness, and healthy controls (15); children with acute viral or bacterial infections (16)	Candidaemia *vs* bacterial *vs* viral infection *vs* SIRS *vs* healthy
**Sweeney7 (28)**	Difference in geometric means between upregulated and downregulated genes, multiplied by ratio of counts of positive to negative genes (bacterial or viral metascore)	Eight cohorts of children and adults with viral and bacterial infections	USA, Australia, UK	Greedy forward search of 72 differentially expressed genes identified by multicohort analysis	24 cohorts of children and adults with viral or bacterial infections, or healthy controls	Viral *vs* bacterial infection
**Trouillet-Assant6 (29)**	Median expression of 6 interferon-stimulated genes (Interferon score (30))	NA	NA	Differential expression using 15 preselected interferon-stimulated genes	Febrile children with bacterial or viral infection	Viral *vs* bacterial infection in febrile children
**Tsalik33 (15)**	Logistic regression (Viral ARI classifier)§	Children and adults with viral, bacterial, or non-infectious acute respiratory illness, and healthy controls	USA	LASSO regression analysis using the 40% of microarray probes with the largest variance after batch correction	Five cohorts of children or adults with viral, bacterial, or non-infectious respiratory illness, or viral or bacterial co-infection	Viral *vs* bacterial acute respiratory illness
**Xu2 (31)**	Logistic regression§	Children and adults with acute febrile illness with confirmed bacterial infection, viral infection	China	Support vector machine learning to identify optimal combination of four candidate transcripts and binary logistic regression modelling	Children and adults with acute febrile illness with confirmed bacterial infection, viral infection, or non-infectious inflammatory disease	Viral *vs* bacterial infection
**Yu3 (8)**	Mean expression (non-RSV infections *vs* controls)	Children with acute respiratory illness and a positive result for a viral infection on a nasopharyngeal swab	USA	Modified supervised principal component analysis using all expressed transcripts	Children with RSV or rhinovirus infection	Viral *vs* healthy in children

**Zaas48 (18)**	Probit regression (Viral classifier)§	Two cohorts of adults challenged with influenza A H3N2 or H1N1	USA	Elastic net using 48 selected genes comprised of: 29 derived as a signature in a previous study (11) , seven shown to be downregulated in analysis of influenza challenge time course data (32) and 12 control genes	Adults presenting to the emergency department with fever and healthy controls	Viral vs bacterial acute respiratory illness

Signatures are referred to by combining the first author's name of the corresponding publication as a prefix, with number of constituent genes as a suffix.

Log_2-transformed transcripts per million data used to calculate all signatures.

*Study by McClain *et al* sought to validate a 36-transcript signature for detection of respiratory viral infections. Model coefficients for the 36-transcript model are not provided; we therefore included the two best performing single transcripts from the study in the current analysis, since they demonstrated similar performance to the full model in the original publication.

¶Where applicable, the name of the signature from the original publication is indicated in brackets.

§Logistic and probit regression models were calculated on the linear predictor scale using model coefficients from original publications.

#IFI27 is also identified as a marker of viral infection by Yu *et al* 2019 (8)

‡3 of 19 genes for this signature were missing from the RNAseq data (MT-RNR2, AC015912.3, SNHG8). The signature was calculated without these.

Acronyms: RSV= respiratory syncytial virus. PAM= prediction analysis of microarrays. LASSO=Least Absolute Shrinkage Selector Operator. RT-LAMP= Reverse Transcription Loop-mediated Isothermal Amplification. NA= not applicable. ATAC-seq= assay for transposase-accessible chromatin with sequencing. SAM= Significant Analysis of Microarray. LOSO= leave-one-study-out. SIRS= Systemic inflammatory response syndrome

Supplementary Figure 1

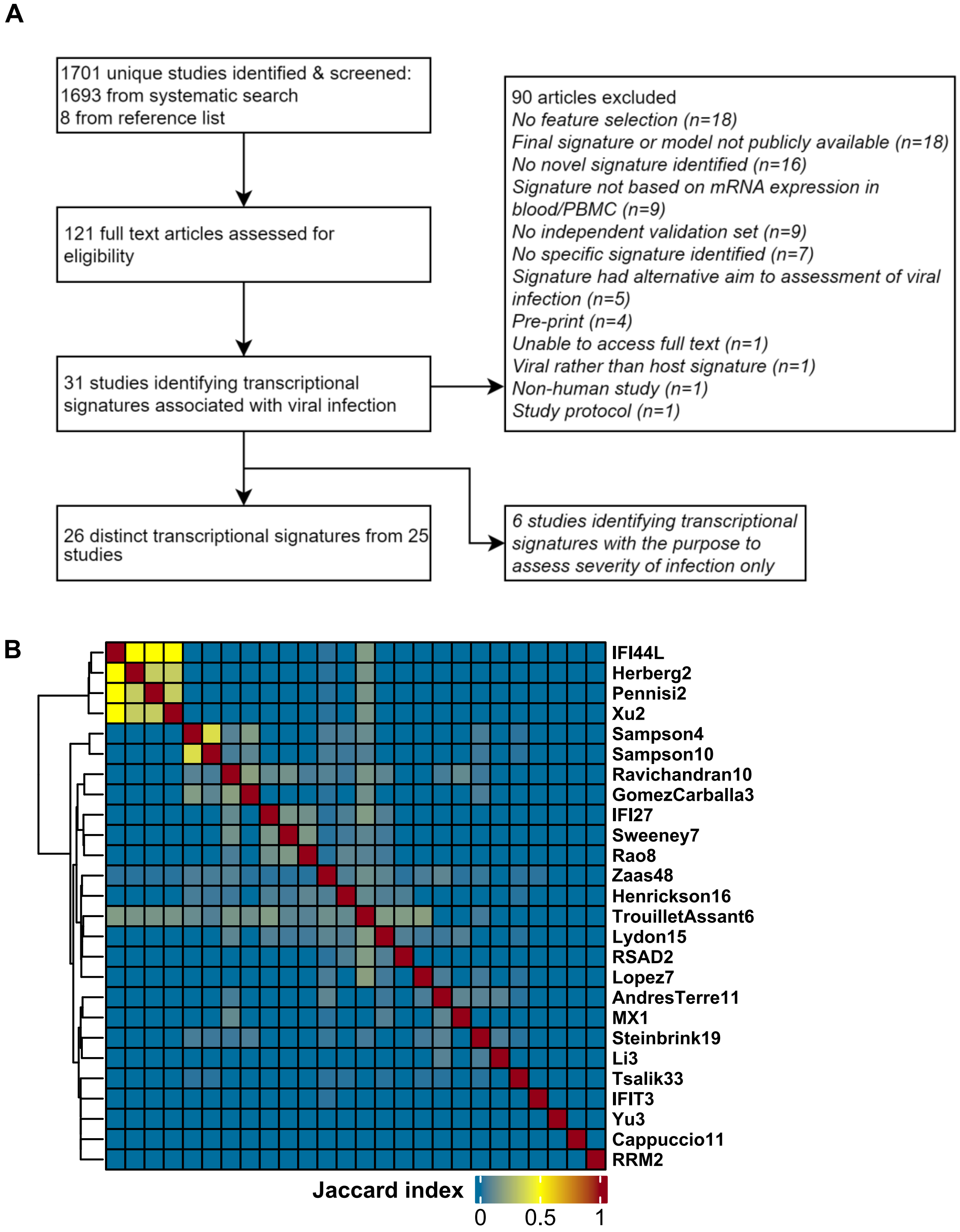

Identification of blood transcriptional biomarkers of viral infection by systematic review and comparison of their gene composition.

(A) Consort diagram of systematic review for identification of blood transcriptional biomarkers of viral infection. **(B)** Jaccard index pairwise comparisons of gene composition for each blood transcriptional signature in a symmetrical matrix with complete linkage clustering.

Supplementary Figure 2

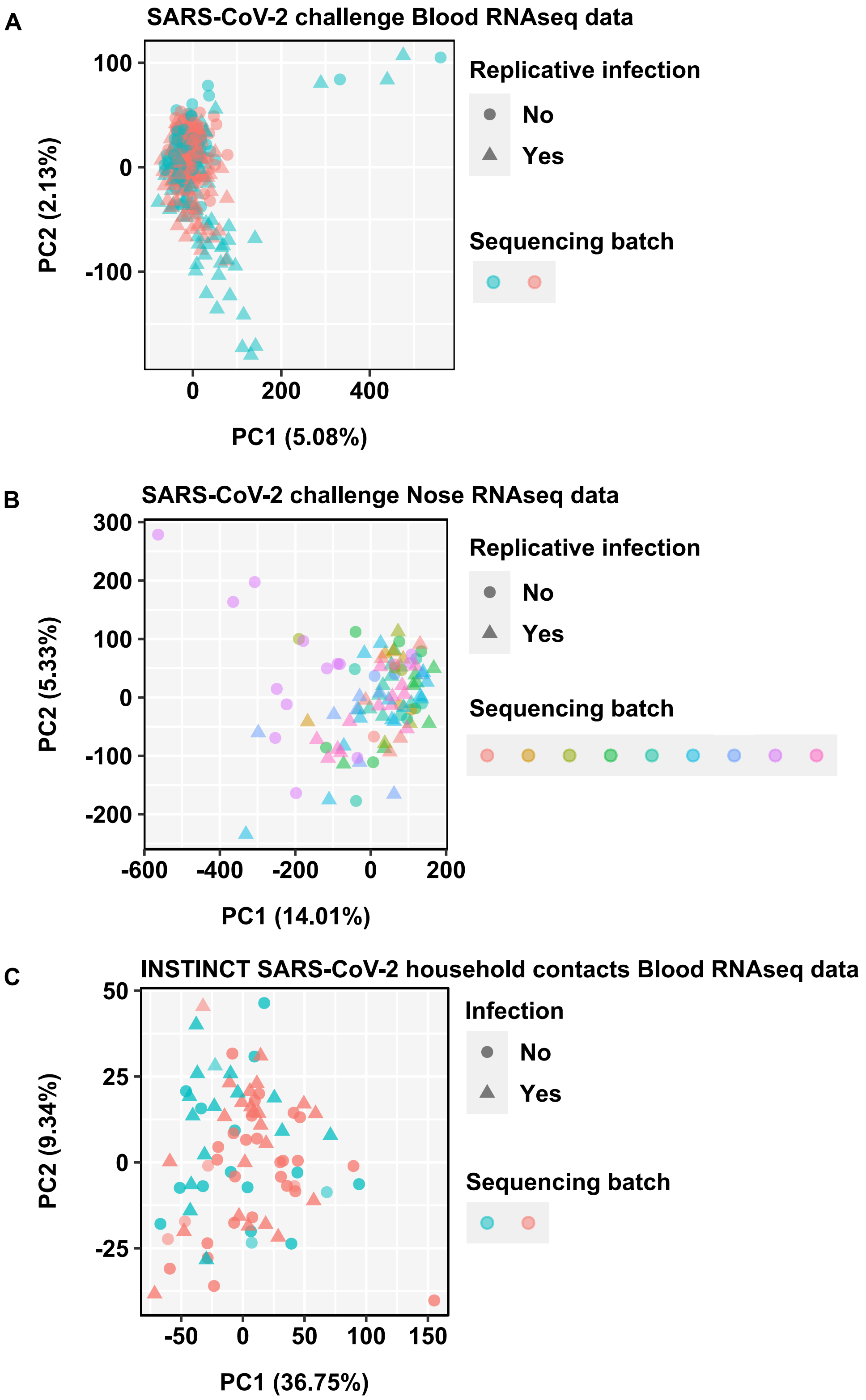

Principal component analysis of RNA sequencing data

Principal component analysis of **(A)** blood and **(B)** nose RNA sequencing data from the SARA-CoV-2 challenge model, and **(C)** blood RNA sequencing data from the INSTINCT SARS-CoV-2 household contacts study, stratified by replicative infection and/or sample processing batch.

Supplementary Figure 3

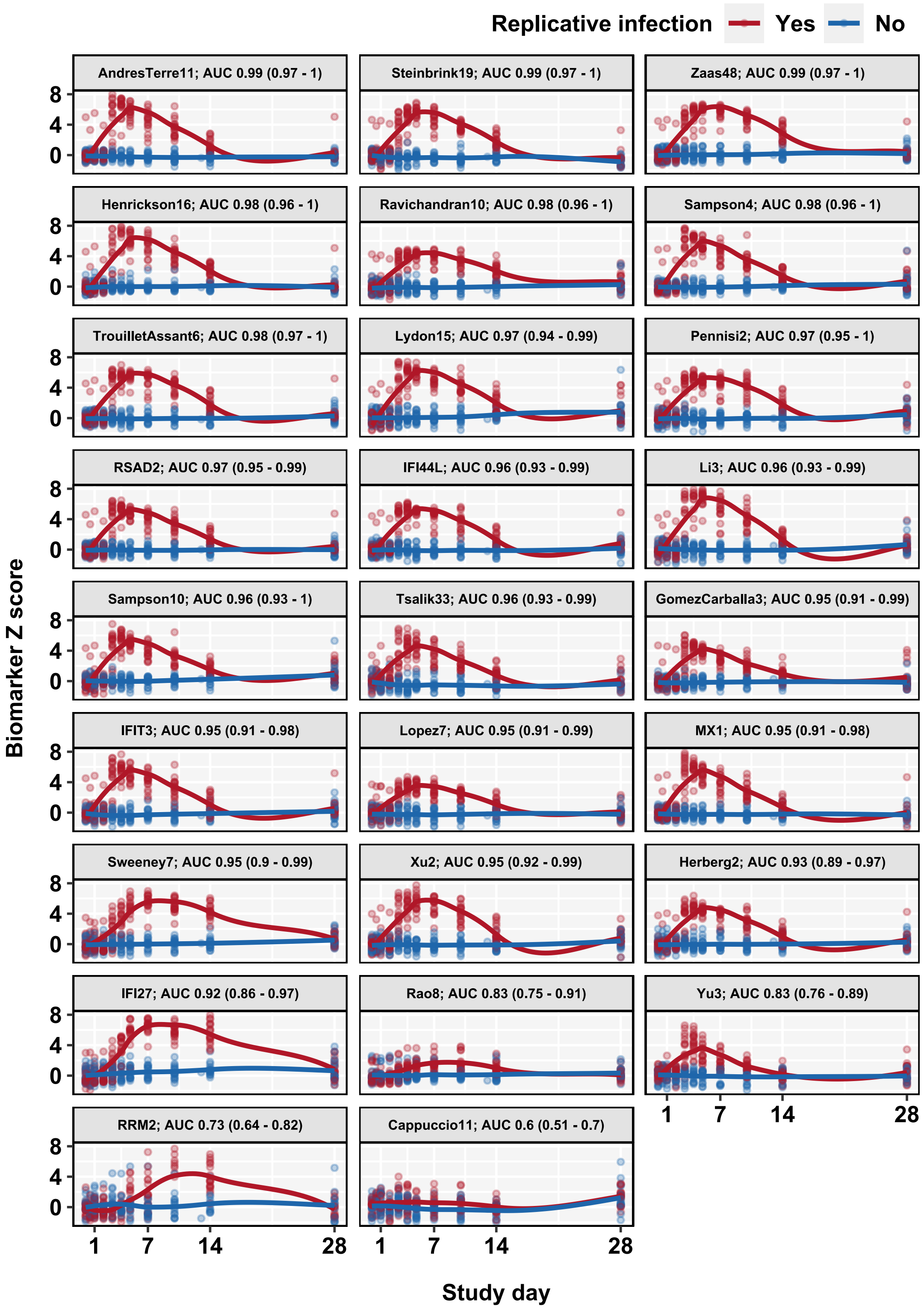

Temporal profiles of blood transcriptional signature scores in participants with and without replicative SARS-CoV-2 infection.

Individual (data points) and loess smoothed summary (line ±95% CI) for standardised blood transcript levels of each blood transcriptional signature in sequential time points after challenge, ranked in descending order of AUROC (±95% CI) for discrimination of participants with (N=17) and without (N=16) replicative viral infection using data from day 3, 7, 10 and 14.

Supplementary Figure 4

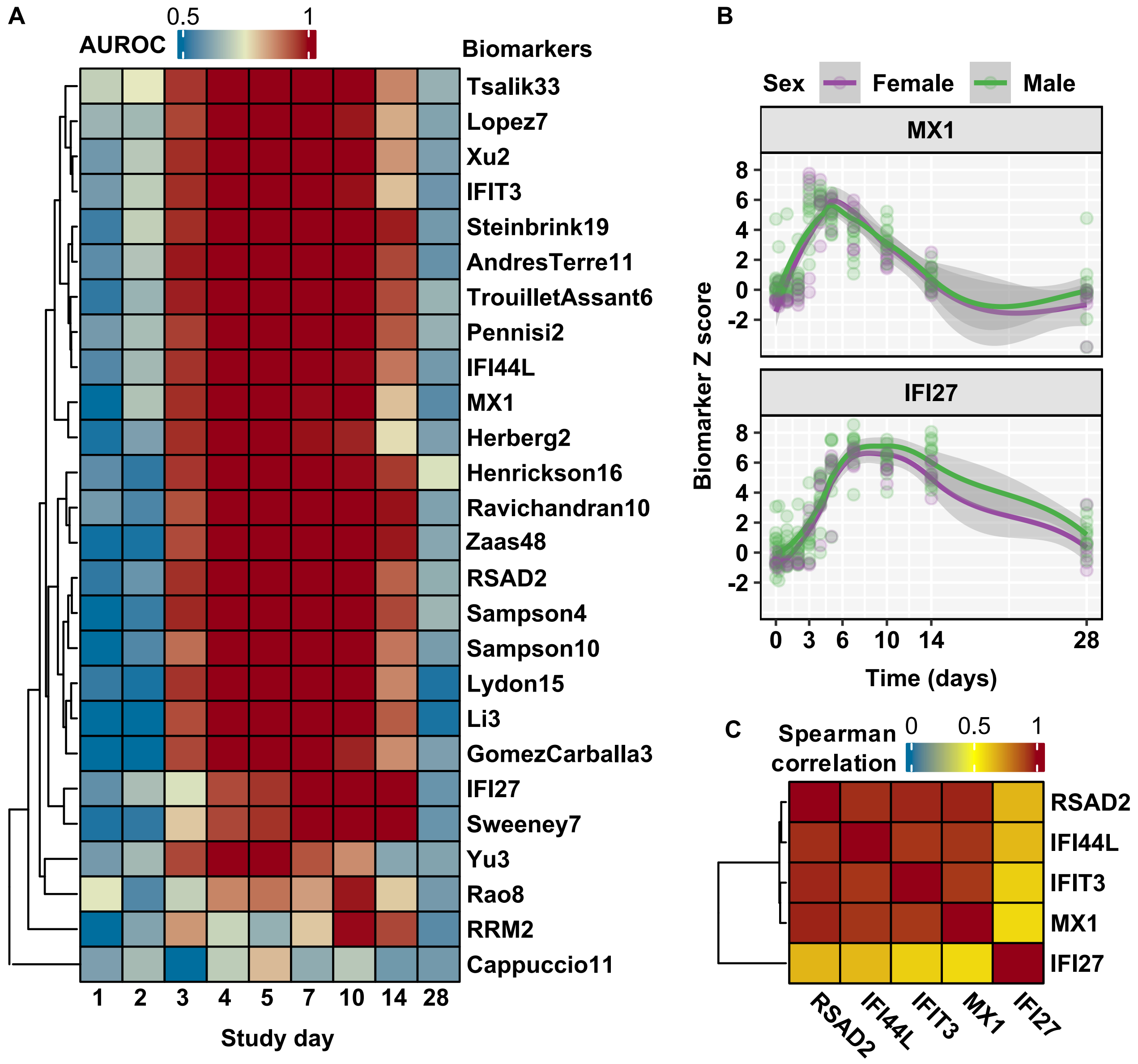

Discrimination of participants with and without replicative SARS-CoV-2 infection by blood transcriptional biomarkers of viral infection.

(A) Heatmap of AUROC point estimates for discrimination of participants with and without replicative SARS-CoV-2 infection by each blood transcriptional signature (rows) stratified by study day (columns). **(B)** Individual (data points) and loess smoothed summary (line ±95% CI) for standardised blood transcript levels of *MX1* and *IFI27* in sequential time points after challenge, for male (N=11) and female (N=6) participants with replicative viral infection. **(C)** Spearman correlation of selected interferon stimulated single gene blood transcriptional biomarker scores across all time points in participants with replicative SARS-CoV-2 infection (N=17).

Supplementary Figure 5.

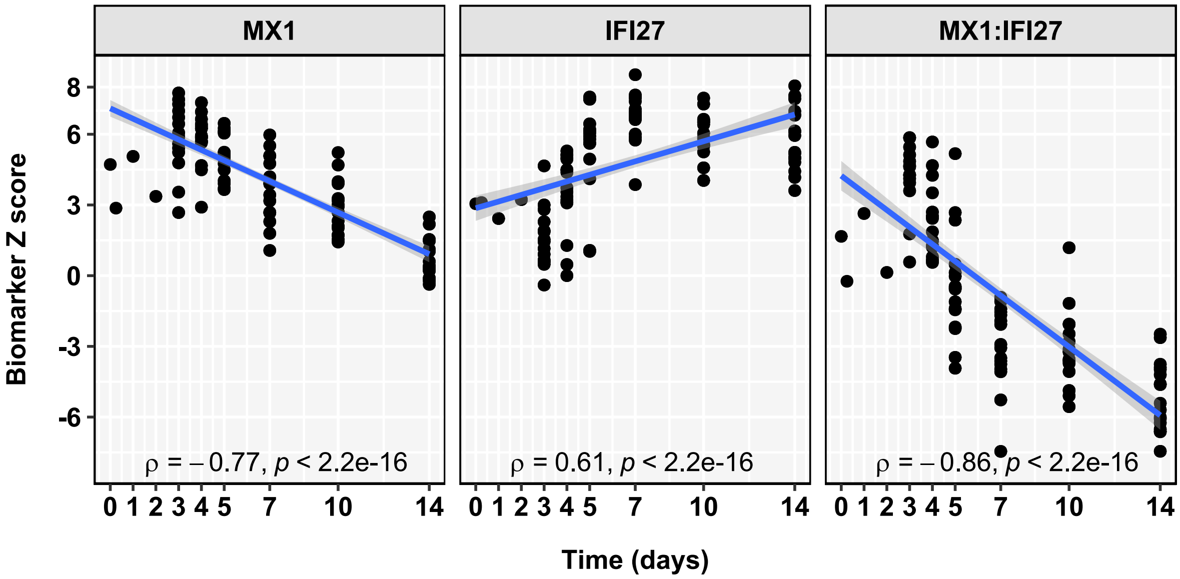

Relationship of MX1 and IFI27 blood RNA levels with time from virus challenge in replicative SARS-CoV-2 infection.

Individual (data points, N=17 subjects per time point) and linear model (line ±95% CI) for standardised blood transcript levels of *MX1*, *IFI27* and *MX1*:*IFI27* ratio in sequential time points after SARS-CoV-2 virus challenge among participants with replicative viral infection, showing 2-sided Spearman rank correlation coefficient (rho) and p value.

Supplementary Figure 6

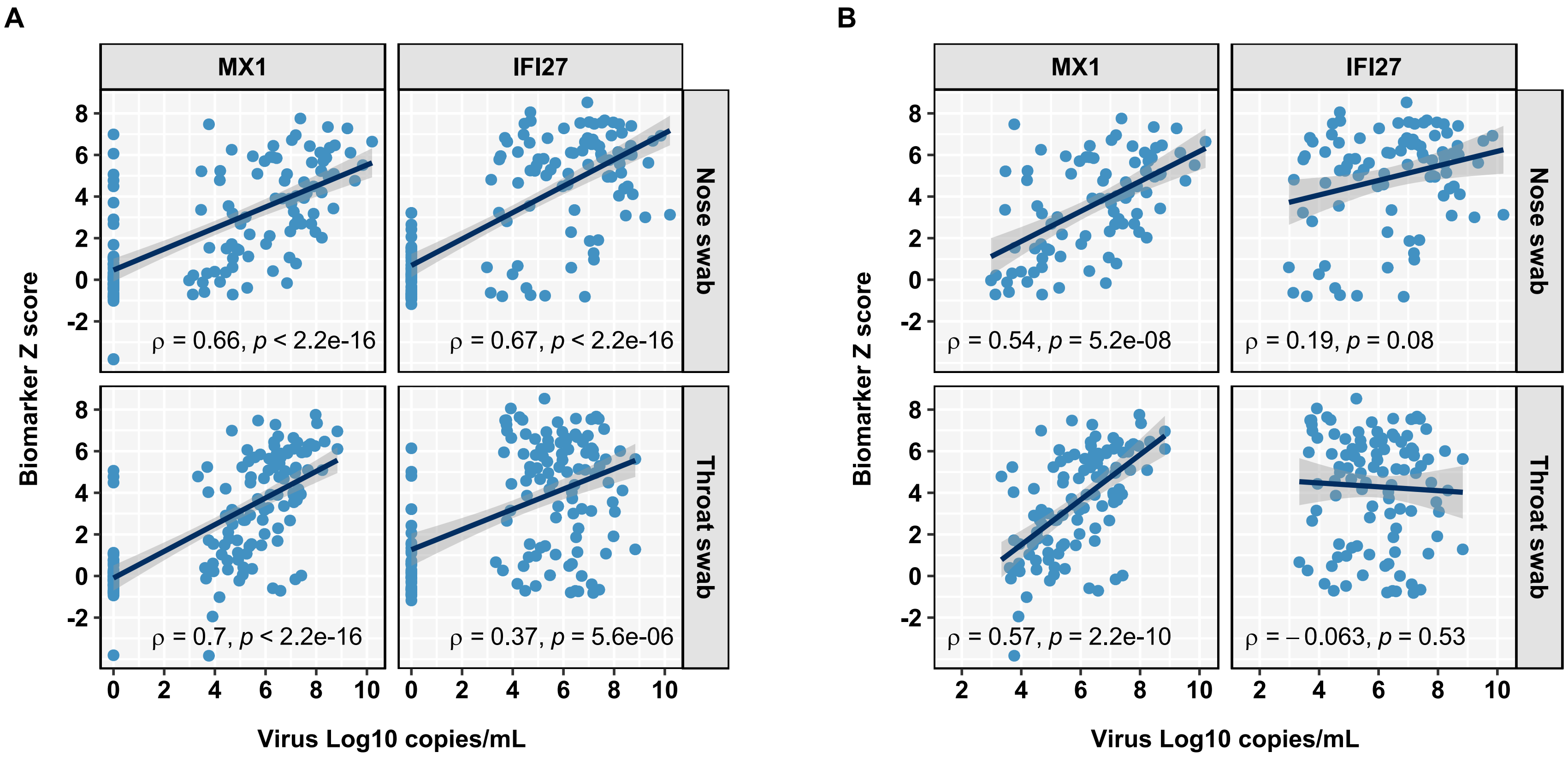

Blood MX1 and IFI27 blood transcript correlation with PCR viral load measurements.

Scatter plot and Spearman correlations of blood *MX1* or *IFI27* transcript levels with nose or throat PCR viral load measurements in participants with replicative SARS-CoV-2 infection (N=17) for all sampling time points **(A)** and for time points with detectable virus by PCR **(B)**, showing 2-sided Spearman rank correlation coefficient (rho) and p value.

Supplementary Figure 7

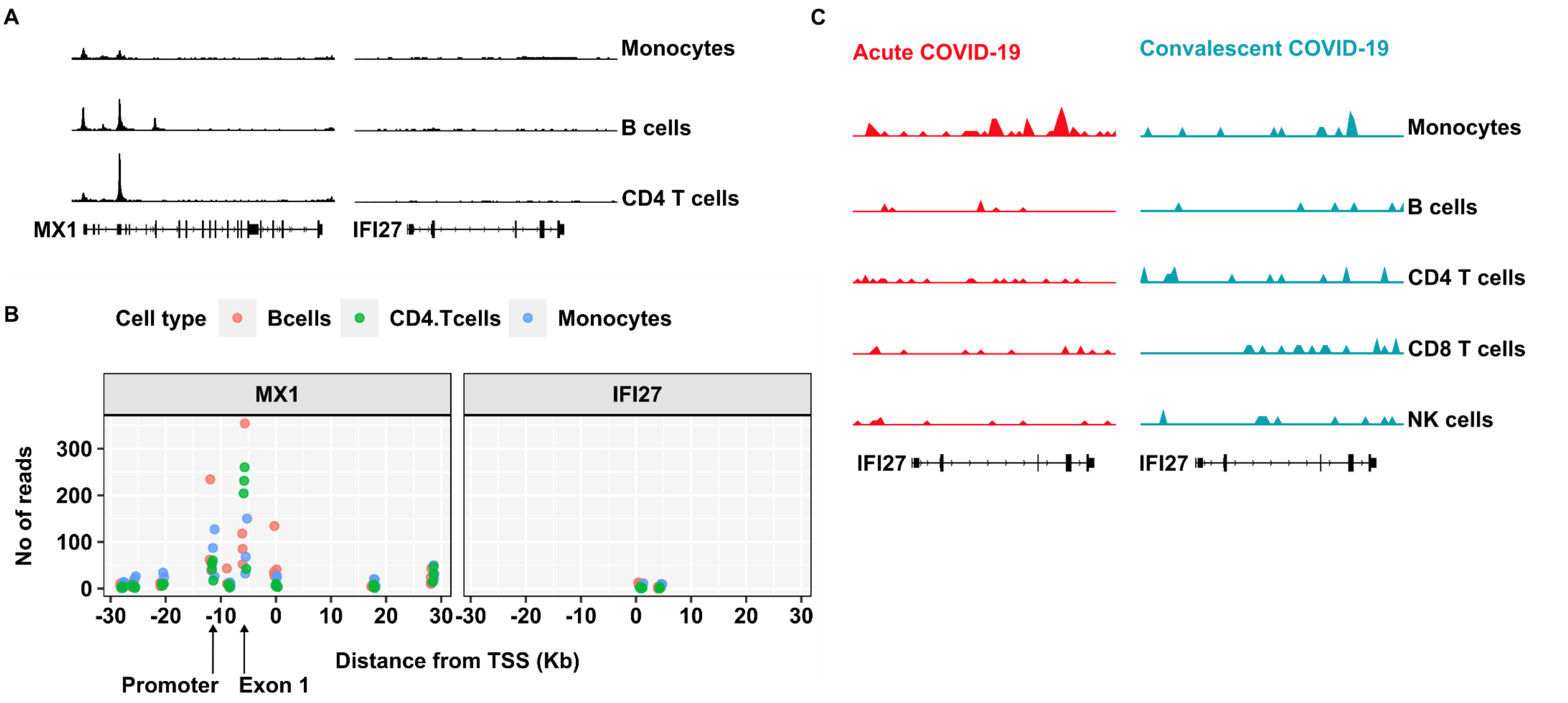

ATAC sequencing reads at MX1 and IFI27 locus in unstimulated peripheral blood mononuclear cells.

(A) Merged ATACseq read tracks (normalised ATAC signal range 0-1.8) from healthy donor sorted unstimulated monocytes (donor N=3), B-cells (N=4) and CD4 T effector cells (N=4) and **(B)** read counts are shown on the Y-axis, with genetic distance between the relevant peak and the gene's transcription start site (TSS) shown on the X-axis. (**C**) Merged single cell ATACseq read tracks at the *IFI27* locus (normalised ATAC signal range 0-0.04) for cell types indicated, in acute and convalescent samples from patients admitted to hospital with COVID-19 (N=8).

Supplementary Figure 8.

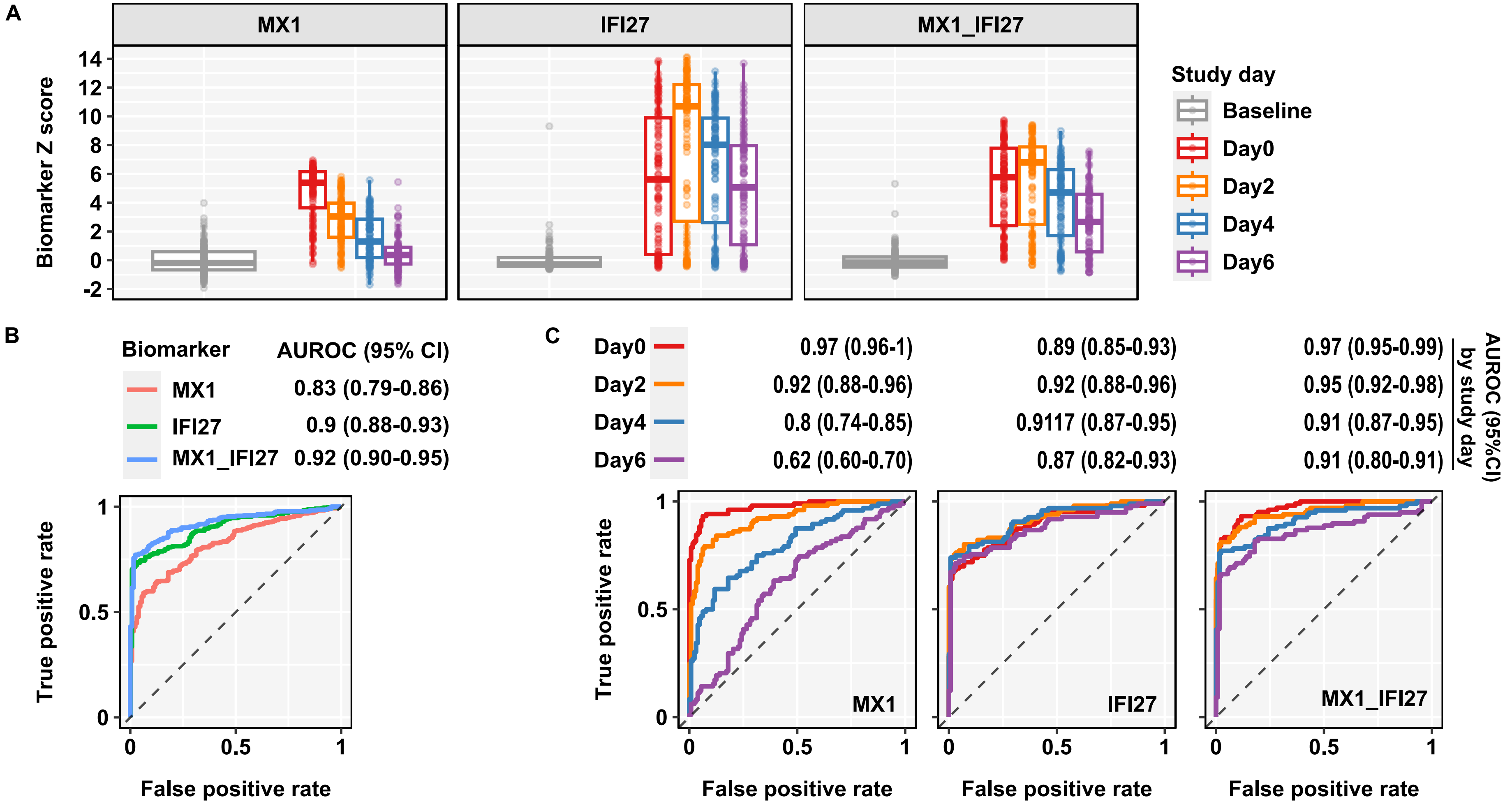

Blood transcript discrimination of post infection time points from pre-infection samples in in unselected community acquired respiratory virus infections from GSE68310.

(A) Individual data points and box plot summaries of blood transcript levels of *MX1*, *IFI27* and average of *MX1* and *IFI27* in pre-infection baseline samples (N=128) and sequential time points on alternate days from study day 0 (up to 48 hours after onset of symptoms) in unselected community acquired respiratory virus infections (N=102-106). **(B)** Receiver operating curve discrimination of all post-infection samples from pre-infection baseline samples by blood transcript levels of *MX1*, *IFI27* and average of *MX1* and *IFI27* with AUROC point estimates (±95% CI). **(C)** Receiver operating curve discrimination of post-infection samples stratified by study day from pre-infection baseline samples by blood transcript levels of *MX1*, *IFI27* and average of *MX1* and *IFI27* with AUROC point estimates (±95% CI).

References

1. Andres-Terre M, McGuire HM, Pouliot Y, Bongen E, Sweeney TE, Tato CM, et al. Integrated, Multi-cohort Analysis Identifies Conserved Transcriptional Signatures across Multiple Respiratory Viruses. Immunity. 2015 Dec 15;43(6):1199.

2. Cappuccio A, Chawla DG, Chen X, Rubenstein AB, Cheng WS, Mao W, et al. Multi-objective optimization identifies a specific and interpretable COVID-19 host response signature. Cell Syst. 2022 Dec 21;13(12):989-1001.e8.

3. Gómez-Carballa A, Barral-Arca R, Cebey-López M, Bello X, Pardo-Seco J, Martinón-Torres F, et al. Identification of a Minimal 3-Transcript Signature to Differentiate Viral from Bacterial Infection from Best Genome-Wide Host RNA Biomarkers: A Multi-Cohort Analysis. Int J Mol Sci. 2021 Jan;22(6):3148.

4. Hoggart CJ. PReMS: Parallel Regularised Regression Model Search for sparse bio-signature discovery [Internet]. bioRxiv; 2018 [cited 2023 Mar 30]. p. 355479. Available from: https://www.biorxiv.org/content/10.1101/355479v3

5. Henrickson SE, Manne S, Dolfi DV, Mansfield KD, Parkhouse K, Mistry RD, et al. Genomic Circuitry Underlying Immunological Response to Pediatric Acute Respiratory Infection. Cell Rep. 2018 Jan 9;22(2):411–26.

6. Herberg JA, Kaforou M, Wright VJ, Shailes H, Eleftherohorinou H, Hoggart CJ, et al. Diagnostic Test Accuracy of a 2-Transcript Host RNA Signature for Discriminating Bacterial vs Viral Infection in Febrile Children. JAMA. 2016 Aug 23;316(8):835–45.

7. Tang BM, Shojaei M, Parnell GP, Huang S, Nalos M, Teoh S, et al. A novel immune biomarker IFI27 discriminates between influenza and bacteria in patients with suspected respiratory infection. Eur Respir J. 2017 Jun;49(6):1602098.

8. Yu J, Peterson DR, Baran AM, Bhattacharya S, Wylie TN, Falsey AR, et al. Host Gene Expression in Nose and Blood for the Diagnosis of Viral Respiratory Infection. J Infect Dis. 2019 Mar 15;219(7):1151–61.

9. Gómez-Carballa A, Cebey-López M, Pardo-Seco J, Barral-Arca R, Rivero-Calle I, Pischedda S, et al. A qPCR expression assay of IFI44L gene differentiates viral from bacterial infections in febrile children. Sci Rep. 2019 Aug 13;9(1):1–12.

10. McClain MT, Constantine FJ, Nicholson BP, Nichols M, Burke TW, Henao R, et al. A blood-based host gene expression assay for early detection of respiratory viral infection: an index-cluster prospective cohort study. Lancet Infect Dis [Internet]. 2020 Sep 24 [cited 2020 Oct 23];0(0). Available from: https://www.thelancet.com/journals/laninf/article/PIIS1473-3099(20)30486-2/abstract

11. Zaas AK, Chen M, Varkey J, Veldman T, Hero AO, Lucas J, et al. Gene Expression Signatures Diagnose Influenza and Other Symptomatic Respiratory Viral Infections in Humans. Cell Host Microbe. 2009 Sep 17;6(3):207–17.

12. Li HK, Kaforou M, Rodriguez-Manzano J, Channon-Wells S, Moniri A, Habgood-Coote D, et al. Discovery and validation of a three-gene signature to distinguish COVID-19 and other viral infections in emergency infectious disease presentations: a case-control and observational cohort study. Lancet Microbe. 2021 Nov 1;2(11):e594–603.

13. lachlancoin. lachlancoin/fspls [Internet]. 2021 [cited 2023 Mar 30]. Available from: https://github.com/lachlancoin/fspls

14. Lopez R, Wang R, Seelig G. A molecular multi-gene classifier for disease diagnostics. Nat Chem. 2018 Jul;10(7):746–54.

15. Tsalik EL, Henao R, Nichols M, Burke T, Ko ER, McClain MT, et al. Host gene expression classifiers diagnose acute respiratory illness etiology. Sci Transl Med. 2016 Jan 20;8(322):322ra11-322ra11.

16. Ramilo O, Allman W, Chung W, Mejias A, Ardura M, Glaser C, et al. Gene expression patterns in blood leukocytes discriminate patients with acute infections. Blood. 2007 Mar 1;109(5):2066–77.

17. Lydon EC, Henao R, Burke TW, Aydin M, Nicholson BP, Glickman SW, et al. Validation of a host response test to distinguish bacterial and viral respiratory infection. EBioMedicine. 2019 Oct 1;48:453–61.

18. Zaas AK, Burke T, Chen M, McClain M, Nicholson B, Veldman T, et al. A Host-Based RT-PCR Gene Expression Signature to Identify Acute Respiratory Viral Infection. Sci Transl Med. 2013 Sep 18;5(203):203ra126-203ra126.

19. Roers A, Hochkeppel HK, Horisberger MA, Hovanessian A, Haller O. MxA Gene Expression after Live Virus Vaccination: A Sensitive Marker for Endogenous Type I Interferon. J Infect Dis. 1994 Apr 1;169(4):807–13.

20. Pennisi I, Rodriguez-Manzano J, Moniri A, Kaforou M, Herberg JA, Levin M, et al. Translation of a host blood RNA Signature distinguishing bacterial from viral infection into a platform suitable for development as a point-of-care test. JAMA Paediatr. 2020 Jan 1;In press.

21. Rao AM, Popper SJ, Gupta S, Davong V, Vaidya K, Chanthongthip A, et al. A robust host-response-based signature distinguishes bacterial and viral infections across diverse global populations. Cell Rep Med [Internet]. 2022 Dec 20 [cited 2023 Mar 30];3(12). Available from: https://www.cell.com/cell-reports-medicine/abstract/S2666-3791(22)00406-2

22. Ravichandran S, Banerjee U, Dr GD, Kandukuru R, Thakur C, Chakravortty D, et al. VB10, a new blood biomarker for differential diagnosis and recovery monitoring of acute viral and bacterial infections. eBioMedicine [Internet]. 2021 May 1 [cited 2023 Mar 30];67. Available from: https://www.thelancet.com/journals/ebiom/article/PIIS2352-3964(21)00145-6/fulltext

23. Samy A, Maher MA, Abdelsalam NA, Badr E. SARS-CoV-2 potential drugs, drug targets, and biomarkers: a viral-host interaction network-based analysis. Sci Rep. 2022 Jul 13;12(1):11934.

24. Overmyer KA, Shishkova E, Miller IJ, Balnis J, Bernstein MN, Peters-Clarke TM, et al. Large-Scale Multi-omic Analysis of COVID-19 Severity. Cell Syst. 2021 Jan 20;12(1):23-40.e7.

25. Sampson D, Yager TD, Fox B, Shallcross L, McHugh L, Seldon T, et al. Blood transcriptomic discrimination of bacterial and viral infections in the emergency department: a multi-cohort observational validation study. BMC Med. 2020 Jul 21;18(1):185.

26. Sampson DL, Fox BA, Yager TD, Bhide S, Cermelli S, McHugh LC, et al. A Four-Biomarker Blood Signature Discriminates Systemic Inflammation Due to Viral Infection Versus Other Etiologies. Sci Rep. 2017 Jun 6;7(1):1–17.

27. Steinbrink JM, Myers RA, Hua K, Johnson MD, Seidelman JL, Tsalik EL, et al. The host transcriptional response to Candidemia is dominated by neutrophil activation and heme biosynthesis and supports novel diagnostic approaches. Genome Med. 2021 Jul 5;13(1):108.

28. Sweeney TE, Wong HR, Khatri P. Robust classification of bacterial and viral infections via integrated host gene expression diagnostics. Sci Transl Med. 2016 Jul 6;8(346):346ra91-346ra91.

29. Trouillet-Assant S, Viel S, Ouziel A, Boisselier L, Rebaud P, Basmaci R, et al. Type I Interferon in Children with Viral or Bacterial Infections. Clin Chem. 2020 Jun 1;66(6):802–8.

30. Pescarmona R, Belot A, Villard M, Besson L, Lopez J, Mosnier I, et al. Comparison of RT-qPCR and Nanostring in the measurement of blood interferon response for the diagnosis of type I interferonopathies. Cytokine. 2019;113:446–52.

31. Xu N, Hao F, Dong X, Yao Y, Guan Y, Yang L, et al. A two-transcript biomarker of host classifier genes for discrimination of bacterial from viral infection in acute febrile illness: a multicentre discovery and validation study. Lancet Digit Health. 2021 Aug 1;3(8):e507–16.

32. Huang Y, Zaas AK, Rao A, Dobigeon N, Woolf PJ, Veldman T, et al. Temporal dynamics of host molecular responses differentiate symptomatic and asymptomatic influenza a infection. PLoS Genet. 2011 Aug;7(8):e1002234.
