# Supplementary material for: SARS-CoV-2 human challenge reveals biomarkers that discriminate early and late phases of respiratory viral infections
Source: Nat Commun. 2024 Nov 30;15:10434. doi: 10.1038/s41467-024-54764-3 (PMC11608262; doi:10.1038/s41467-024-54764-3)
Supplement: Supplementary file 3 — Description of Additional Supplementary Files [file 41467_2024_54764_MOESM3_ESM.docx]

Description of Additional Supplementary Files

**File Name:** Supplementary Data 1

**Description:** Summary of the full text systematic review of the literature to identify blood transcriptional signatures of acute viral infection.
