# Supplementary material for: SARS-CoV-2 human challenge reveals biomarkers that discriminate early and late phases of respiratory viral infections
Source: Nat Commun. 2024 Nov 30;15:10434. doi: 10.1038/s41467-024-54764-3 (PMC11608262; doi:10.1038/s41467-024-54764-3)
Supplement: Supplementary file 5 — Reporting Summary [file 41467_2024_54764_MOESM5_ESM.pdf]

nature portfolio

Corresponding author(s): Mahdad Noursadeghi

Last updated by author(s): Nov 4, 2023

Reporting Summary

Nature Portfolio wishes to improve the reproducibility of the work that we publish. This form provides structure for consistency and transparency in reporting. For further information on Nature Portfolio policies, see our Editorial Policies and the Editorial Policy Checklist.

Statistics

For all statistical analyses, confirm that the following items are present in the figure legend, table legend, main text, or Methods section.

n/a	Confirmed	
☐	☒	The exact sample size (*n*) for each experimental group/condition, given as a discrete number and unit of measurement
☐	☒	A statement on whether measurements were taken from distinct samples or whether the same sample was measured repeatedly
☐	☒	The statistical test(s) used AND whether they are one- or two-sided<br>*Only common tests should be described solely by name; describe more complex techniques in the Methods section.*
☒	☐	A description of all covariates tested
☐	☒	A description of any assumptions or corrections, such as tests of normality and adjustment for multiple comparisons
☐	☒	A full description of the statistical parameters including central tendency (e.g. means) or other basic estimates (e.g. regression coefficient) AND variation (e.g. standard deviation) or associated estimates of uncertainty (e.g. confidence intervals)
☐	☒	For null hypothesis testing, the test statistic (e.g. *F*, *t*, *r*) with confidence intervals, effect sizes, degrees of freedom and *P* value noted<br>*Give P values as exact values whenever suitable.*
☒	☐	For Bayesian analysis, information on the choice of priors and Markov chain Monte Carlo settings
☒	☐	For hierarchical and complex designs, identification of the appropriate level for tests and full reporting of outcomes
☒	☐	Estimates of effect sizes (e.g. Cohen's *d*, Pearson's *r*), indicating how they were calculated

Our web collection on statistics for biologists contains articles on many of the points above.

Software and code

Policy information about availability of computer code

Data collection	No software was used to collect the data.
Data analysis	Analyses were performed in R (version 4.2.2). Custom code for deriving RNA signature Z scores from a gene expression matrix is available on Github: https://github.com/JRosenheim/SARS-CoV-2_challenge/blob/main/Viral_biomarker_scores.R (DOI: 10.5281/zenodo.10021757). Area under the receiver operating characteristic curve analysis was performed using the pROC R package. Spearman rank correlation coefficients were calculated using the ggpur R package. Graphs were plotted using the ggplot2 R package.

For manuscripts utilizing custom algorithms or software that are central to the research but not yet described in published literature, software must be made available to editors and reviewers. We strongly encourage code deposition in a community repository (e.g. GitHub). See the Nature Portfolio guidelines for submitting code & software for further information.

Data

Policy information about availability of data

All manuscripts must include a data availability statement. This statement should provide the following information, where applicable:

- Accession codes, unique identifiers, or web links for publicly available datasets
- A description of any restrictions on data availability
- For clinical datasets or third party data, please ensure that the statement adheres to our policy

All source data for the analyses presented in this study are provided in Supplementary Data 2. Processed RNAseq data is available at ArrayExpress for the SARS-CoV-2 challenge study (accession number: E-MTAB-12993, https://www.ebi.ac.uk/biostudies/arrayexpress/studies/E-MTAB-12993). To comply with data privacy

restrictions, raw sequencing data is available under managed access through the European Genome-Phenome Archive (https://ega-archive.org), under the following accession numbers: EGAD50000000942 for SARS-CoV-2 challenge study(https://ega-archive.org/datasets/EGAD50000000942), EGAD50000000956 for Influenza H3N2 challenge study (https://ega-archive.org/datasets/EGAD50000000956), and EGAD50000000684 for the INSTINCT SARS-CoV-2 household contact study https://ega-archive.org/datasets/EGAD50000000684). Data will be shared with investigators whose proposed use is within the scope of participant consent subject to a data access agreement.
RNAseq data are also available from Gene Expression Omnibus (GEO) under the following accession numbers: GSE73072 (https://www.ncbi.nlm.nih.gov/geo/query/acc.cgi?acc=GSE73072) for previous human challenge studies, and GSE68310 (https://www.ncbi.nlm.nih.gov/geo/query/acc.cgi?acc=GSE68310) for previous community acquired respiratory virus infections. ATACseq data are available from GEO under accession number GSE118189 (https://www.ncbi.nlm.nih.gov/geo/query/acc.cgi?acc=GSE118189) for unstimulated PBMC, and the European Phenome Genome Archive under accession number EGAD00001007963 (https://ega-archive.org/datasets/EGAD00001007931) for the COMBAT consortium data.

Research involving human participants, their data, or biological material

Policy information about studies with human participants or human data. See also policy information about sex, gender (identity/presentation), and sexual orientation and race, ethnicity and racism.

Reporting on sex and gender	Male and female participants were included in this study. Sex was determined by self-reporting. The two human challenge studies comprised 55 participants in total, of which 22 were female. Where applicable results have been reported stratified by sex. The main finding of the study (namely differential temporal profile of MX1 andIFI27 responses to replicative SARS-CoV-2 infection) is comparable in results stratified by sex.
Reporting on race, ethnicity, or other socially relevant groupings	In the SARS-CoV-2 human challenge model, participnts had an age range 18-29 years, 22% were of female sex, and 90% had White or Caucasian ancestry.<br>In the INSTINCT SARS-CoV-2 household contact study, participants had an age range 7-79 years, 48% were of female sex and had 90% White ancestry.<br>In the Influenza H3N2 human challenge, participants had an age range 22-55 years, 50% were of female sex, and 68% White, 32% Black or Asian ancestry.
Population characteristics	Summary level data on participant characteristics are provided in the methods narrative.
Recruitment	Participant recruitment for each of the study cohorts presented is reported in the original manuscripts cited.
Ethics oversight	Regulatory approvals for the human studies presented herein were provided by the UK Health Research Authority under the following reference numbers: 20/UK/2001 and 20/UK/0002 for the SARS-CoV-2 challenge study; 20/NW/0231 for the INSTINCT study; 19/LO/1441 for the H3N2 influenza challenge study.

Note that full information on the approval of the study protocol must also be provided in the manuscript.

Field-specific reporting

Please select the one below that is the best fit for your research. If you are not sure, read the appropriate sections before making your selection.

☒ Life sciences ☐ Behavioural & social sciences ☐ Ecological, evolutionary & environmental sciences

For a reference copy of the document with all sections, see nature.com/documents/nr-reporting-summary-flat.pdf

Life sciences study design

All studies must disclose on these points even when the disclosure is negative.

Sample size	The present study did not form the basis of sample size calculations for any of the study cohorts presented. The sample size for the analysis in the present manuscript was determined by the availability of blood and nose RNA data from each study cohort.
Data exclusions	There were no data exclusions. All available data were included in the analyses.
Replication	All analyses included data from multiple participants. Analysis of SARS-CoV-2 challenge was conducted in 35 participants. Analysis of Influenza challenge was conducted in 20 particpants. Analysis of INSTINCT SARS-CoV-2 household contact study involved 52 participants. Analysis of community acquired respiratory infection included 128 participants. All results were reported with 95% confidence intervals. These conclusions presented were replicated in each of these four independent study cohorts.
Randomization	There was no a priori allocation of participants to separate groups. Between group analyses was based on presence or absence of replicative virus infection.
Blinding	Sample processing for transcriptional profiling was blinded to virological and clinical metadata.

Reporting for specific materials, systems and methods

We require information from authors about some types of materials, experimental systems and methods used in many studies. Here, indicate whether each material, system or method listed is relevant to your study. If you are not sure if a list item applies to your research, read the appropriate section before selecting a response.

Materials & experimental systems

n/a	Involved in the study
☒	☐ Antibodies
☐	☒ Eukaryotic cell lines
☒	☐ Palaeontology and archaeology
☒	☐ Animals and other organisms
☒	☐ Clinical data
☒	☐ Dual use research of concern
☒	☐ Plants

Methods

n/a	Involved in the study
☒	☐ ChIP-seq
☒	☐ Flow cytometry
☒	☐ MRI-based neuroimaging

Eukaryotic cell lines

Policy information about cell lines and Sex and Gender in Research

Cell line source(s)	Vero (WHO) cells purchased from the European collection of authenticated cell cultures (ECACC, catalogue no 88020401)
Authentication	Vero cells were authenticated by STR profile analysis.
Mycoplasma contamination	Vero cells were tested negative for Mycoplasma by culture isolation, Hoescht DNA staining and PCR.
Commonly misidentified lines (See ICLAC register)	No commonly misidentified cell lines were used in this study.
